# A New Dental Inhaler

**Published:** 1877-06

**Authors:** 


### ARTICLE X.

### *A New Dental Inhaler.*

The following is an extract from a letter to the Galvano-Faradic Manufacturing Co., 167 East 37th St., from Dr. D. S. Oliphant, of Toronto, Canada:

"I am tempted to mention a device of mine for use of chloroform. By its use I recently successfully chloroformed a patient in a dental office with only three quarters of one dram of chloroform, when previously, in the ordinary way, I had used *Three ounces.* I made an Inhaler out of a child's rubber band comb—bending the ends and fastening them together thus—O. Over the top of this oval I tie a piece of muslin, or linen, so that the interstics of the teeth of the comb are left open. The comb serves as a framework to keep the chloroform from touching the skin. I hang it over nose and open mouth, and rapidly drop 6 or 8 drops on the muslin diaphragm, after which *one drop per second* is quite sufficient to keep up supply of vapor. The operator, skillful dentist, could hardly believe his senses when he saw how small a quantity had sufficed, and how easily the patient (a nervous woman) breathed during the whole operation. He extracted three teeth—one upper molar and two eye teeth. If you think my device either good or new communicate it to whom it may concern or benefit."—*Physicians Monitor.*

---

